# Xiao Cheng Qi Decoction, an Ancient Chinese Herbal Mixture, Relieves Loperamide-Induced Slow-Transit Constipation in Mice: An Action Mediated by Gut Microbiota

**DOI:** 10.3390/ph17020153

**Published:** 2024-01-24

**Authors:** Amanguli Tuohongerbieke, Huaiyou Wang, Jiahui Wu, Zhengqi Wang, Tingxia Dong, Yamiao Huang, Dequan Zhu, Dongmei Sun, Karl Wah Keung Tsim

**Affiliations:** 1 Shenzhen Key Laboratory of Edible and Medicinal Bioresources, HKUST Shenzhen Research Institute, Shenzhen 518057, China; amanguli@ust.hk (A.T.); wanghy707@163.com (H.W.); jwuct@connect.ust.hk (J.W.); botina@ust.hk (T.D.); ymhuanga@ust.hk (Y.H.); 2 State Key Laboratory of Antiviral Drugs, School of Pharmacy, Henan University, Kaifeng 475004, China; 3 Division of Life Science and Center for Chinese Medicine, The Hong Kong University of Science and Technology, Hong Kong, China; zwangie@connect.ust.hk; 4 Guangdong Efong Pharmaceutical Co., Ltd., Guangdong Provincial Key Laboratory of Traditional Chinese Medicine Formula Granule, Foshan 528244, China; 13068614346@163.com (D.Z.); dongmeisun2019@163.com (D.S.)

**Keywords:** slow transit constipation, microbe, short-chain fatty acids, Chinese medicine

## Abstract

Xiao Cheng Qi (XCQ) decoction, an ancient Chinese herbal mixture, has been used in treating slow-transit constipation (STC) for years. The underlying action mechanism in relieving the clinical symptoms is unclear. Several lines of evidence point to a strong link between constipation and gut microbiota. Short-chain fatty acids (SCFAs) and microbial metabolites have been shown to affect 5-HT synthesis by activating the GPR43 receptor localized on intestinal enterochromaffin cells, since 5-HT receptors are known to influence colonic peristalsis. The objective of this study was to evaluate the efficacy of XCQ in alleviating clinical symptoms in a mouse model of STC induced by loperamide. The application of loperamide leads to a decrease in intestinal transport and fecal water, which is used to establish the animal model of STC. In addition, the relationship between constipation and gut microbiota was determined. The herbal materials, composed of Rhei Radix et Rhizoma (Rhizomes of *Rheum palmatum* L., Polygonaceae) 55.2 g, Magnoliae Officinalis Cortex (Barks of *Magnolia officinalis* Rehd. et Wils, Magnoliaceae) 27.6 g, and Aurantii Fructus Immaturus (Fruitlet of *Citrus aurantium* L., Rutaceae) 36.0 g, were extracted with water to prepare the XCQ decoction. The constipated mice were induced with loperamide (10 mg/kg/day), and then treated with an oral dose of XCQ herbal extract (2.0, 4.0, and 8.0 g/kg/day) two times a day. Mosapride was administered as a positive drug. In loperamide-induced STC mice, the therapeutic parameters of XCQ-treated mice were determined, i.e., (i) symptoms of constipation, composition of gut microbiota, and amount of short-chain fatty acids in feces; (ii) plasma level of 5-HT; and (iii) expressions of the GPR43 and 5-HT4 receptor in colon. XCQ ameliorated the constipation symptoms of loperamide-induced STC mice. In gut microbiota, the treatment of XCQ in STC mice increased the relative abundances of *Lactobacillus*, *Prevotellaceae_UCG_001*, *Prevotellaceae_NK3B31_group*, *Muribaculaceae*, and *Roseburia* in feces and decreased the relative abundances of *Desulfovibrio*, *Tuzzerella*, and *Lachnospiraceae_ NK4A136_group*. The levels of SCFAs in stools from the STC group were significantly lower than those the control group, and were greatly elevated via treatment with XCQ. Compared with the STC group, XCQ increased the plasma level of 5-HT and the colonic expressions of the GPR43 and 5-HT4 receptor, significantly. The underlying mechanism of XCQ in anti-constipation could be related to the modulation of gut microbiota, the increase in SCFAs, the increase in plasma 5-HT, and the colonic expressions of the GPR43 and 5-HT4 receptor. Our results indicate that XCQ is a potent natural product that could be a therapeutic strategy for constipation.

## 1. Introduction

Constipation is a risk factor for many gastrointestinal disorders, with a global prevalence ranging from 14% to 30% of the population [1]. Slow-transit constipation (STC), a type of chronic constipation affecting quality of life, is distinguished by a prolonged bowel transit time, decreased stool frequency, loss of bowel movement, dry and hard stools, and abdominal distension [2]. Therefore, it is urgent to have effective methods to relieve the symptoms of STC. Today, laxatives and catalytic medicines are utilized to treat STC. Although the usage of irritating laxatives has a significant effect, there are patients who apparently have adverse effects and develop drug dependence [3].

Several lines of evidence indicate that gut microbiota play an important role in developing chronic constipation. Disorder of gut microbiota happens during the onset and progression of chronic constipation, as characterized primarily by an increased abundance of *Bacteroides* [4], and a decreased abundance of *Bifidobacteria*, *Lactobacilli* [5], and other butyrate-producing bacteria, e.g., *Faecalibacterium* and *Roseburia* [6]. The metabolism of gut microbiota results in the production of short-chain fatty acids (SCFAs), endotoxins, and other substances that may impact gut motility [7]. The modulation of gut microbiota and the production of SCFAs may be the possible targets in searching for novel anti-STC medications. The neurotransmitter 5-hydroxytryptamine (5-HT) and its receptor, receptor 4 (5-HT4 receptor), are the primary stimulant molecules. Indeed, 5-HT plays a crucial role in controlling the dynamics of the gastrointestinal system [8].

Xiao Cheng Qi (XCQ) decoction is a traditional Chinese herbal formula that was recorded by “Shang Han Lun” around 200 A.D., composed of Rhei Radix et Rhizoma (Rhizomes of *Rheum palmatum* L., Polygonaceae), Magnoliae Officinalis Cortex (Barks of *Magnolia officinalis* Rehd. et Wils, Magnoliaceae), and Aurantii Fructus Immaturus (Fruitlet of *Citrus aurantium* L., Rutaceae). This herbal decoction is frequently used to treat illnesses of the digestive system in clinical practice, including recovery from post-operative gastrointestinal function, intestinal infarction, and constipation after stroke. Each herb in the XCQ decoction plays a specific role in treating constipation. Rhei Radix et Rhizoma is a laxative herb known to promote bowel movements and relieve constipation; this herb is also known to have anti-inflammatory and anti-bacterial properties. In addition, Rhei Radix et Rhizoma can stimulate the secretion of bile and digestive enzymes, aiding in the digestion and absorption of food. Magnoliae Officinalis Cortex is a herb that can promote the movement of Qi (energy) in the body and relieve stagnation. According to Chinese medicinal theory, constipation is often attributed to the stagnation of Qi in the gut, and Magnoliae Officinalis Cortex is able to alleviate this stagnation, thereafter improving gut motility and relieving constipation. Aurantii Fructus Immaturus is a herb that can regulate the Qi in the gut and promote bowel movements, which has a mild laxative effect in relieving constipation, which also reduces abdominal bloating and discomfort, common symptoms of constipation. Overall, the three herbs in XCQ help to regulate gut motility, relieve constipation, reduce inflammation, and promote overall digestive health [9,10]. Due to its precise curative efficacy and recorded clinical application, XCQ has been included in the Catalogue of Ancient Classic Famous Prescriptions (National Administration of Traditional Chinese Medicine (NATCM), 2018). Although XCQ effectively shows treatment in STC and significantly reduces its related symptoms, its underlying mechanism is as yet unknown. 

In XCQ decoction, Rhei Radix et Rhizoma serves as a purgative in helping constipation by promoting the release of colonic mucus from mast cells and by enhancing the microenvironment in the intestine [11]. Sennoside A, an inactive glycoside in Rhei Radix et Rhizoma, is converted into an active metabolite, rheinanthrone, by gut microbiota, which has a purgative effect as well [12]. In a loperamide-induced STC rat model, hesperidin, a flavonoid glycoside of Aurantii Fructus Immaturus, enhanced the gastrointestinal function by boosting the expression of the 5-HT4 receptor [13]. Moreover, Magnoliae Officinalis Cortex has excellent gastrointestinal functions, and comprehensively improves a variety of gut symptoms, e.g., abdominal pain and flatulence [14]. These findings therefore motivated us to determine the role of XCQ in regulating gut microbiota during the therapy of STC. In the loperamide-induced mouse model of STC, the relationship between constipation and gut microbiota, including the profiles of short-chain fatty acids (SCFAs), was determined under the treatment of XCQ.

## 2. Results

### 2.1. Effects of XCQ on Constipation Indices in STC Mice

The herbal extract of XCQ was subject to HPLC analysis. A fingerprint containing thirteen major peaks was generated. By comparing their retention time with the known standards, representative naringin, neohesperidin, aloe-emodin, rhein, honokiol, magnolol, emodin, chrysophanol, and physcion were identified (Figure S1 in Supplementary Materials). A quantitative analysis of XCQ extract was performed, showing the amounts of naringin (~9.84%), neohesperidin (~13.93%), anthraquinones (~0.56%, i.e., aloe-emodin, rhein, emodin, chrysophanol, and physcion), and honokiol and magnolol (~0.32%).

**Figure 1:**
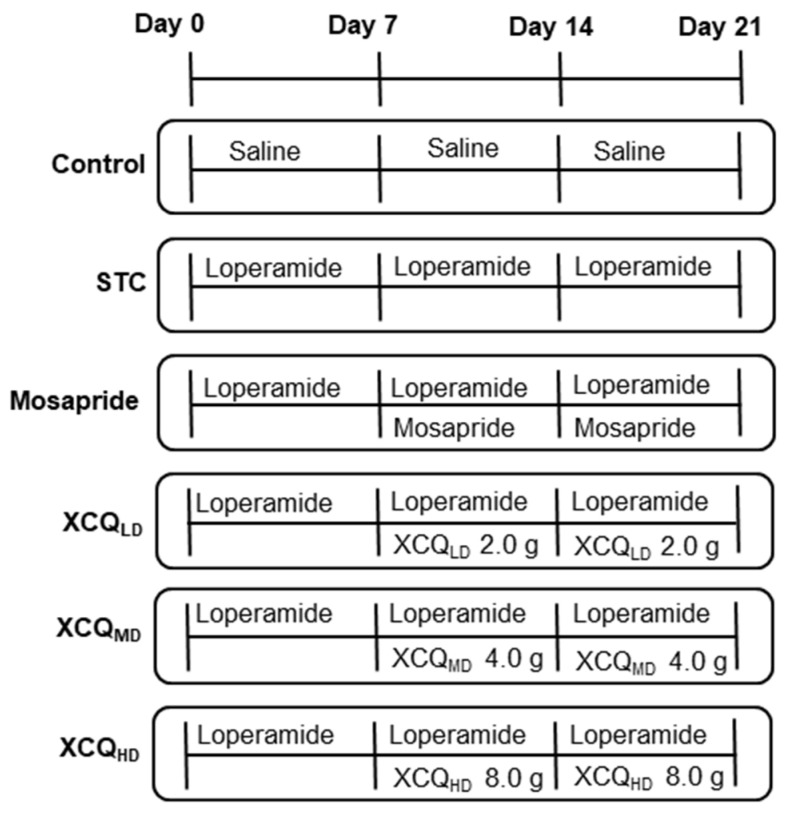
Schematic outline of STC model establishment and XCQ treatment. Mice were divided into six groups: control, normal mice treated with saline; STC, loperamide (10 mg/kg/day)-induced STC mice treated with saline; mosapride, loperamide-induced STC mice treated with mosapride (2.5 mg/kg/day); XCQ_LD_ (low dose of XCQ 2 g/kg/day), XCQ_MD_ (medium dose of XCQ 4 g/kg/day) and XCQ_HD_ (high dose of XCQ 8 g/kg/day), LOP-induced STC mice treated with XCQ herbal extracts. The drug treatments were given via gavage from day 8 to day 21.

**Figure 2:**
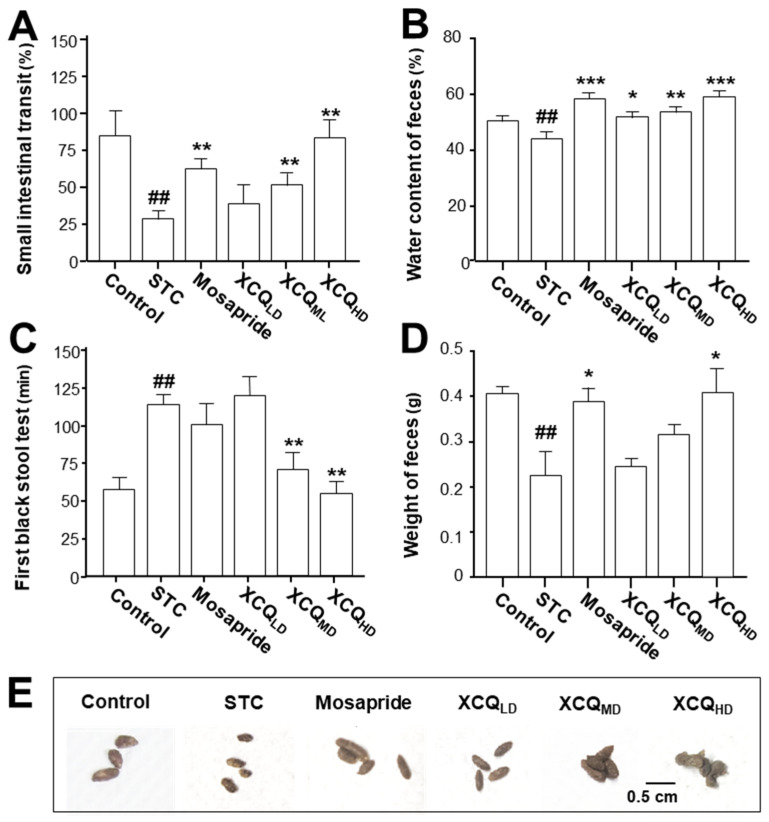
XCQ ameliorates the constipation symptoms in STC mice. The experimental protocol and groups are listed in Figure 1. At 21 days, the mice and their feces were subjected to analysis. (**A**) The percentage of the small intestinal transit rate. (**B**) The content of fecal water content. (**C**) The first black stool defecation time. (**D**) The weight of feces. (**E**) The shape of collected feces. Values are presented as mean ± SEM, *n* = 3–5. Significant differences were assessed using a one-way ANOVA: ## *p* < 0.01 vs. the control group; * *p* < 0.05, ** *p* < 0.01, and *** *p* < 0.001 vs. the STC group.

**Figure 3:**
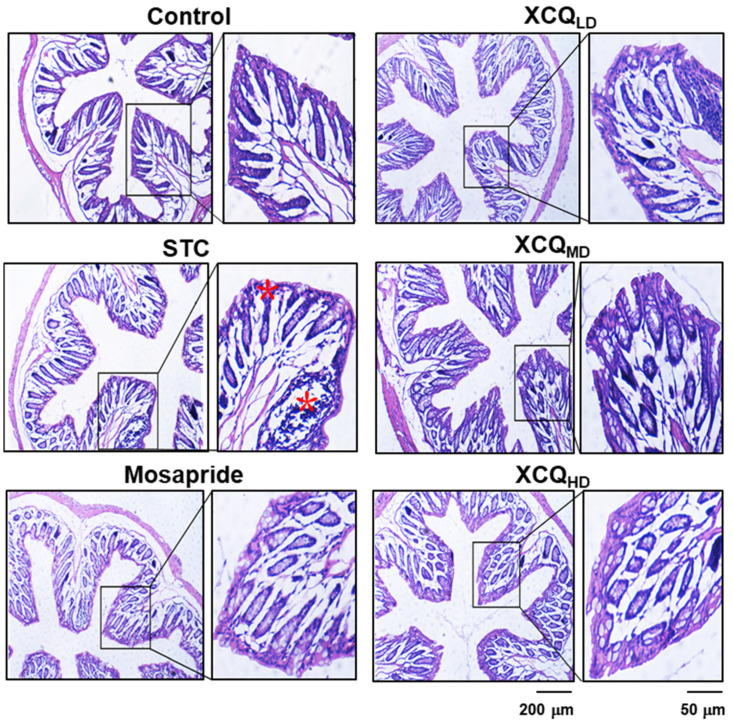
Effects of XCQ on histology of colonic tissue in STC mice. The experimental protocol and groups are listed in Figure 1. At 21 days, the colon samples were subjected to analysis. Colonic tissues were stained via H&E staining. Representative images are shown, *n* = 5. The stars indicate colonic mucosal injury and inflammation in the colon tissues of STC mice.

A mouse STC model was established via an oral gavage administration of loperamide, and thereafter different dosages of XCQ were applied; the experimental outline is shown (Figure 1). The body weights of different treatments showed no significant change (Figure S2A in Supplementary Materials); however, the fecal water content of the STC group was significantly decreased on day 7 after the treatment of loperamide, i.e., a development of the disease model. On day 14, fecal water content was markedly recovered following XCQ treatments in all doses and in the mosapride-treated group (Figure S2B in Supplementary Materials). The STC group showed significantly lower intestinal motility than that of control group (*p* < 0.01), indicating that STC mice were successfully established with the expected symptoms (Figure 2A). The treatments of middle and high doses of XCQ (XCQ_MD_ and XCQ_HD_), or mosapride, effectively improved intestinal motility in STC mice (*p* < 0.01). Moreover, the fecal water content in the STC group showed a significant decrease, as compared to that in the control group (*p* < 0.01) (Figure 2B). In contrast, XCQ administration showed a dose-dependent increase in fecal water content (*p* < 0.01). The STC group took a longer time for the first black stool defecation than did the control group, significantly (*p* < 0.01), while XCQ_MD_ and XCQ_HD_ took a significantly shorter time (*p* < 0.01) (Figure 2C). The weight of feces for a 24 h period on the 21st day was decreased considerably in STC mice (*p* < 0.01), and was significantly increased by XCQ treatments (*p* < 0.05) (Figure 2D). The feces of XCQ-treated mice was moist, shiny, and swollen, as compared to that of the STC group (Figure 2E). These findings indicated that XCQ effectively relieved the constipation indices of loperamide-induced STC mice. In addition, H&E staining was performed to evaluate the effect of XCQ on the structure of the intestinal tissues of loperamide-induced mice, e.g., the breakage and loss of epithelial cells in the mucosal layer, a reduction in cupped cells, inflammatory infiltrations, and the atrophy and decrease of the crypt. XCQ and mosapride treatments improved colonic mucosal injury and inflammation in the colon tissues of STC mice (Figure 3).

### 2.2. XCQ Restores Gut Microbiota Disorder of STC Mice

**Figure 4:**
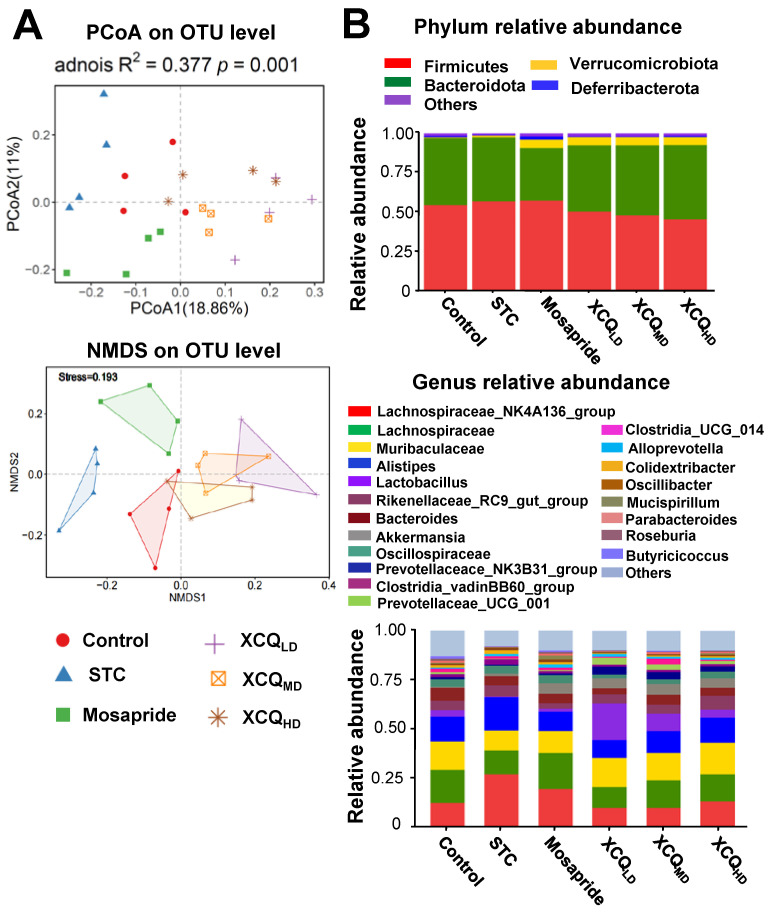
XCQ regulates gut microbiota in STC mice. The experimental protocol and groups are listed in Figure 1. At 21 days, the feces were subjected to analysis. (**A**) PCoA and NMDS analysis based on Bray–Curtis distance shows the β-diversity of each group at the OTU level. (**B**) The community distribution at phylum and genus levels. Each group is represented by different color/symbol combinations, *n* = 4.

The refractive curve of each sample is shown, indicating the sufficient depth of RNA sequencing (Figure S3A,B in Supplementary Materials). The α-diversity analysis showed that both the Shannon and Chao indexes of STC group were less than those of the control group at the OTU level (Figure S3C,D in Supplementary Materials). Mosapride, and medium and high doses of XCQ showed an elevation in Shannon analysis (Figure S3C in Supplementary Materials). PCoA and NMDS methods were employed to determine the β-diversity of each group at the OTU level, where the features of the untreated control and STC group were well distinguished (Figure 4A). In addition, the treatment groups were different from the model group. 

**Figure 5:**
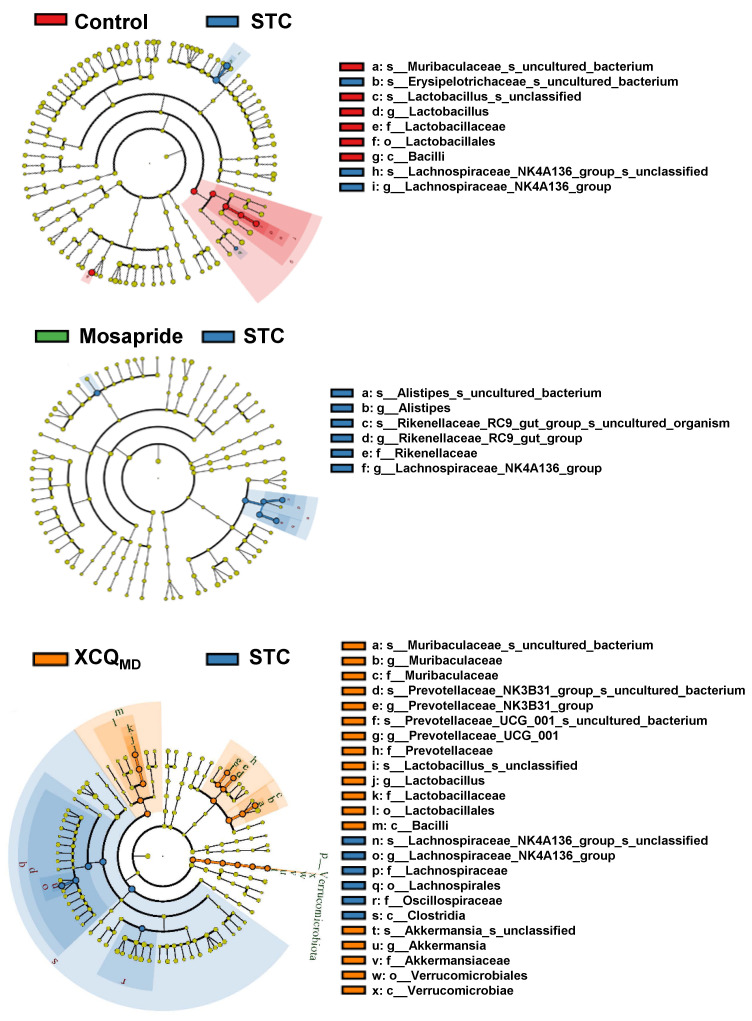
A cladogram of LEfSe analysis shows the differently enriched gut microbiomes based on a comparison with the model groups. LDA score > 4; *n* = 4.

**Figure 6:**
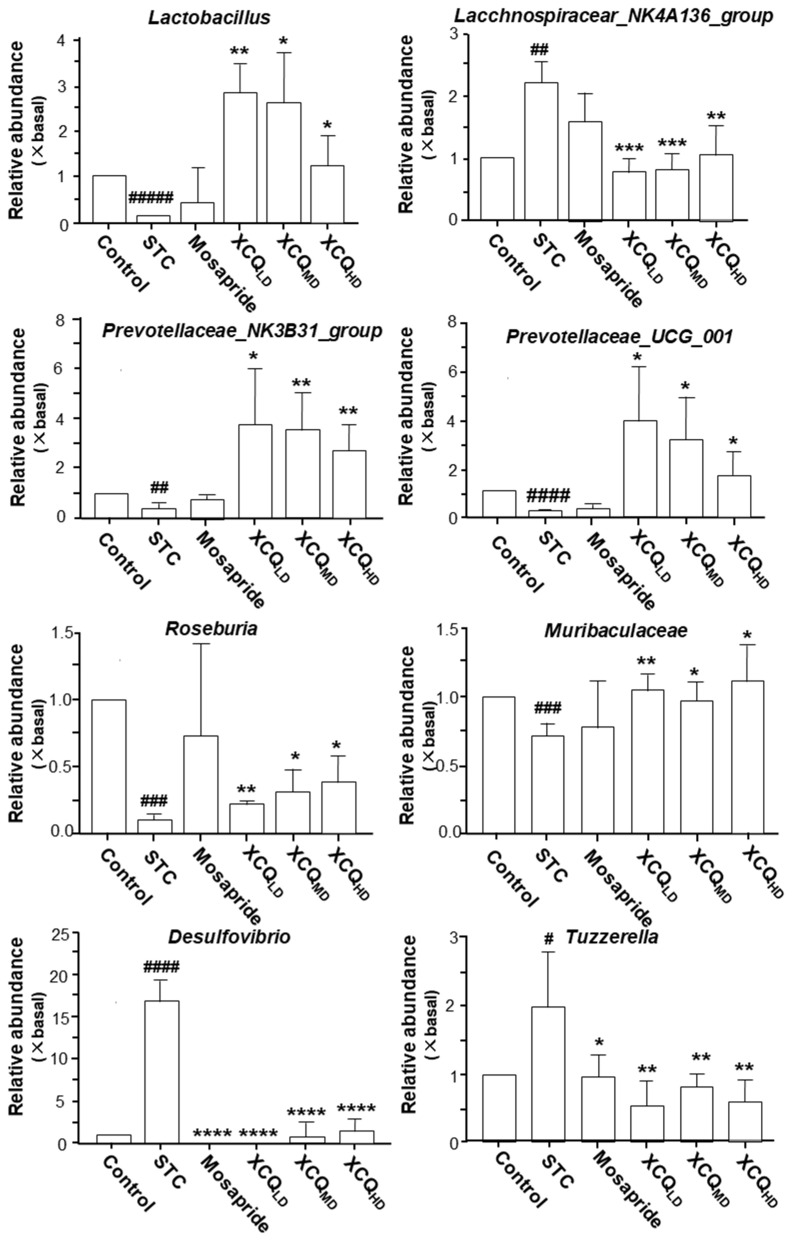
XCQ regulates the composition of microbes in STC mice. The experimental protocol and groups are listed in Figure 1. At 21 days, the feces were subjected to analysis. The relative abundance of different bacterial taxa, as indicated here, was calibrated. The control group’s data were used as the basal values, and the data of other groups were normalized. Values are presented as fold of change (×basal) in mean ± SEM, *n* = 4. Significance differences were assessed via one-way ANOVA: # *p* < 0.05, ## *p* < 0.01, ### *p* < 0.001, #### *p* < 0.0001 and ##### *p* < 0.00001vs. the control group; * *p* < 0.05, ** *p* < 0.01, *** *p* < 0.001, and **** *p* < 0.0001 vs. the STC group.

**Figure 7:**
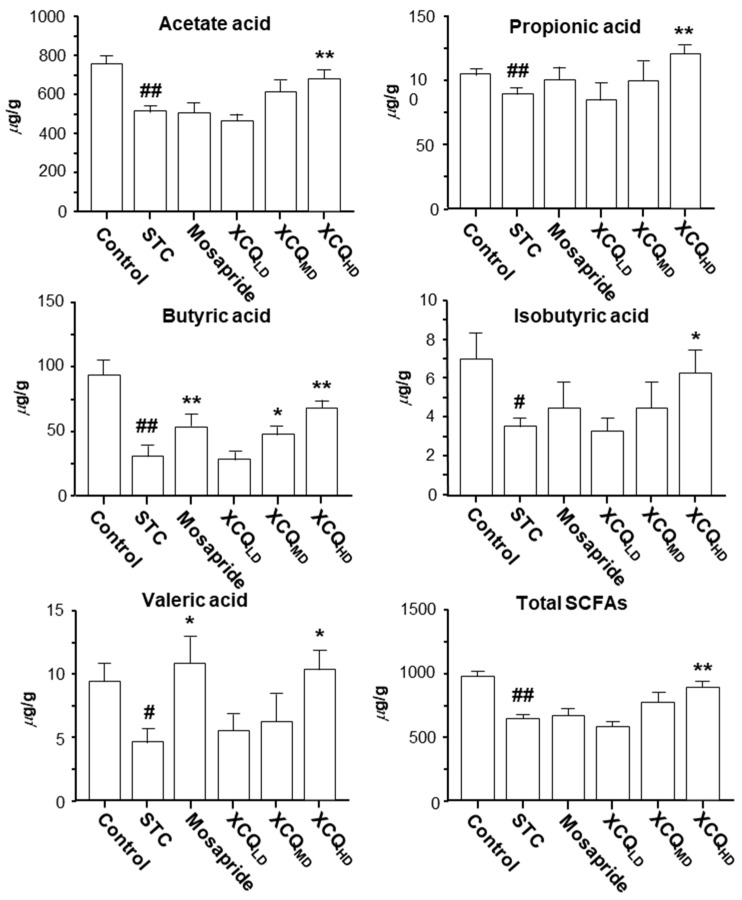
XCQ regulating the levels of SCFAs in stools of STC mice. The experimental protocol and groups are listed in Figure 1. At 21 days, the feces were subjected to analysis. The amounts of acetic acid, propionic acid, butyric acid, isobutyric acid, valeric acid, and total SCFAs in the collected stools were determined via GC-FID. Values are presented as mean ± SEM, *n* = 3. Significance differences were assessed via one-way ANOVA: # *p* < 0.05 and ## *p* < 0.01 vs. the control group; * *p* < 0.05 and ** *p* < 0.01 vs. the STC group.

The community analysis was carried out at phylum and genus levels. *Lachnospiraceae_NK4A136_group*, a dominant genus belonging to the phylum of Firmicutes, was highly enriched in the STC group and was less abundant in other groups (Figure 4B). The increase in the abundance of the Firmicutes phylum in the STC group was due to a significant rise in the *Lachnospiraceae_NK4A136_group* genus from Firmicutes, as compared with that in the other groups. The abundance of Firmicutes in the XCQ treatment group was lower than that in the STC group because the amount of *Lachnospiraceae_NK4A136_group* downregulated in the XCQ treatment group. According to the literature, *Lachnospiraceae_ NK4A136_group* is an indicator of gut dysbiosis, which shows greater abundance during severe gut dysbiosis [15]. The phylum Verrucomicrobia, the third major microorganism in the gut, increased slightly in the model group, as compared to that in the control group, and significantly increased in the mosapride and XCQ groups. The changes in the phyla of the genera *Akkermansia* and *Verrucomicrobiota* were the same. Thus, the changes in the *Verrucomicrobiota* phylum were mainly due to changes in the genus *Akkermansia*. *Akkermansia* is a probiotic that can degrade mucin, improve intestinal barriers, and reduce obesity [16,17]. The increase in the abundance of *Akkermansia* in XCQ and mosapride groups may be related to improvements in gut function and reductions in inflammation. Further differences between the STC group and other groups were compared via LEfSe analysis. Control and XCQ groups contained a higher level of Bacilli, *Lactobacillales*, *Lactobacillaceae*, and *Lactobacillus* at class, order, family, and genus levels, respectively (Figure 5). However, under the same strict FDR threshold at 4, the mosapride group did not show much of a difference from the model group. At the genus level, the relative abundances of *Lactobacillus*, *Prevotellaceae_UCG_001*, *Prevotellaceae_NK3B31_group*, *Muribaculaceae*, and *Roseburia* were markedly reduced in the STC group, as compared with that in control group (Figure 6). The STC-reduced microbial population was elevated via the treatment of XCQ in a dose-dependent manner. Additionally, the STC group showed a higher abundance of *Desulfovibrio*, *Tuzzerella*, and *Lachnospiraceae_NK4A136_group* (Figure 6). These results indicated that the changes in the gut microbial community during STC pathogenesis could be reversed via treatment with XCQ. The levels of major SCFAs in the stool, produced in STC mice, were determined. The levels of acetic acid, propionic acid, isobutyric acid, butyric acid, valeric acid, and total SCFAs in the stools of STC mice were significantly lower than those in the control group. As expected, the STC-reduced SCFAs were significantly elevated via treatment with a high-dose of XCQ (Figure 7).

### 2.3. XCQ Restores the Levels of 5-HT, 5-HT4, and GPR43 Receptors in STC Mice

**Figure 8:**
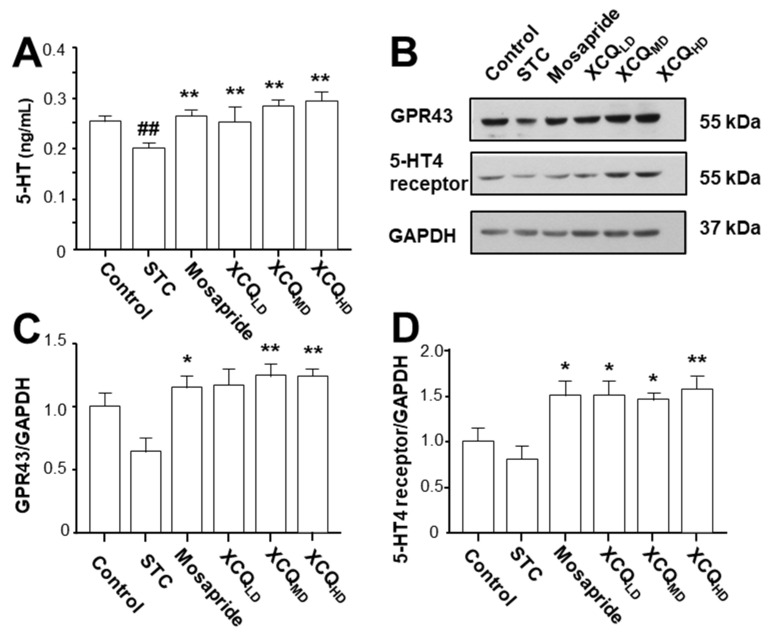
XCQ regulating the level of 5-HT in the plasma and the expression of the GPR43 and 5-HT4 receptor in colon in STC mice. The experimental protocol and groups are listed in Figure 1. At 21 days, the plasma and colon samples were subjected to analysis. (**A**) The plasma level of 5-HT in different groups. (**B**) The relative abundances of the GPR43 (~55 kDa) and 5-HT4 receptor (~55 kDa) colon tissues determined via Western blotting, and a representative gel image. (**C**) The protein expression of GPR43 in the colon. (**D**) The protein expression of the 5-HT4 receptor in colon. Values presented as mean ± SEM, *n* = 3. Significant differences were assessed via one-way ANOVA: ## *p* < 0.01 vs. the control group; * *p* < 0.05 and ** *p* < 0.01 vs. the STC group.

5-HT is a mediator controlling several physiological processes in the gastrointestinal tract, including intestinal secretion and gut motility [18]. The level of 5-HT in plasma was measured via ELISA. The level of 5-HT in STC mice was reduced, as compared to that in the control group (*p* < 0.001), which was significantly recovered having had treatment with various doses of XCQ, as well as mosapride treatment, as compared to STC mice (*p* < 0.01) (Figure 8A). Thus, XCQ promoted the secretion of 5-HT in the plasma. 

**Figure 9:**
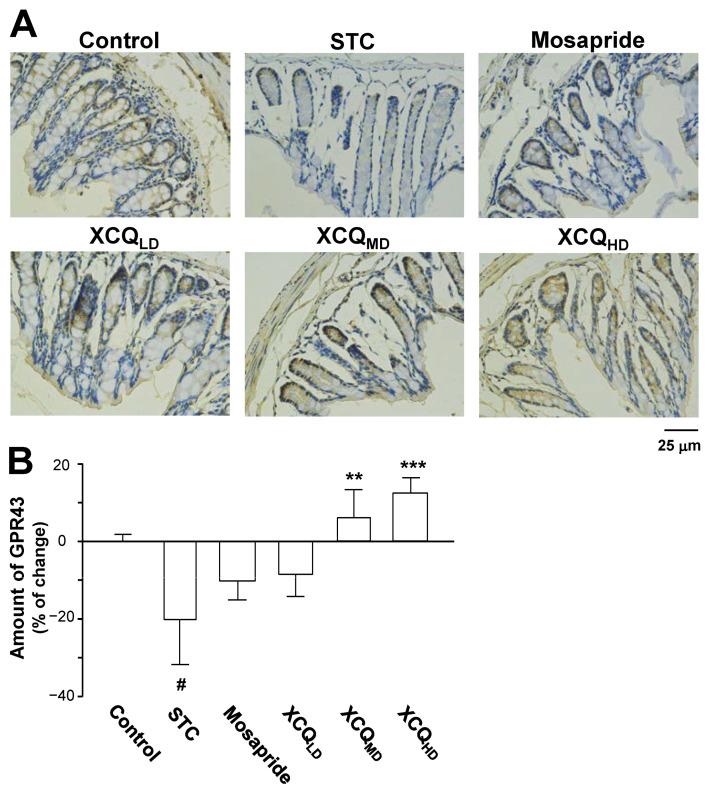
XCQ regulates the expression of GPR43 in the colon tissues of STC mice. The experimental protocol and groups are listed in Figure 1. At 21 days, the colon samples were subjected to analysis. (**A**) Immunohistochemical analysis of colonic sections from different groups. Representative images are shown, *n* = 4. (**B**) Quantification of the immunostaining of the GPR43 receptor in the colonic tissues of different groups using Image-Pro Plus software (Media Cybernetics). Values are presented as a percentage of the change in mean ± SEM, *n* = 5. Significance differences were assessed via one-way ANOVA: # *p* < 0.05, vs. the control group; ** *p* < 0.01, and *** *p* < 0.001 vs. the STC group.

**Figure 10:**
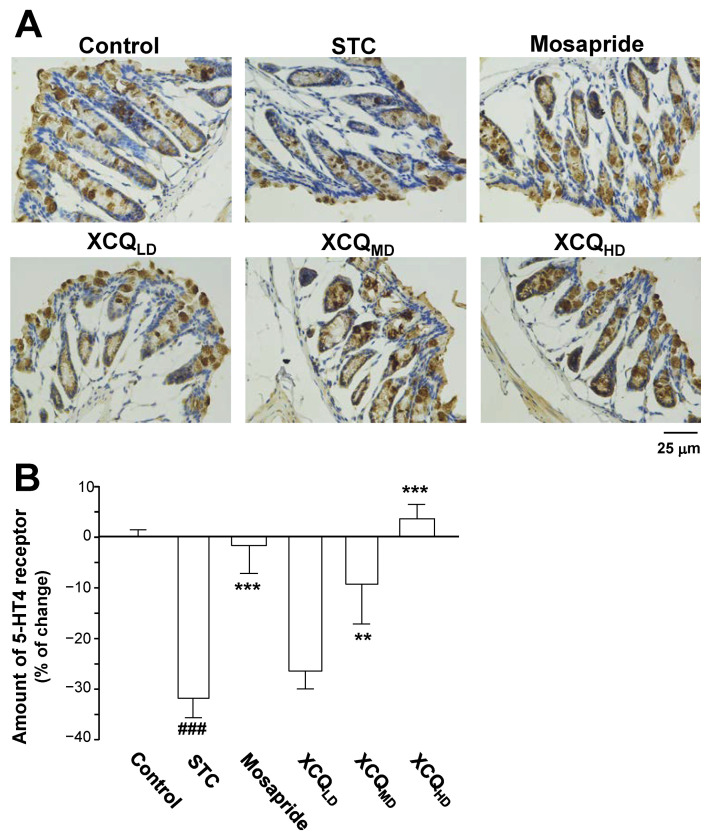
XCQ regulates the expression of the 5-HT4 receptor in the colon tissues of STC mice. The experimental protocol and groups are listed in Figure 1. At 21 days, the colon samples were subjected to analysis. (**A**) Immunohistochemical analysis of colonic sections from different groups. Representative images are shown, *n* = 4. (**B**) Quantification of the immunostaining of the 5-HT4 receptor in the colonic tissues of different groups using Image-Pro Plus software (Media Cybernetics). Values are presented as a percentage of the change in mean ± SEM, *n* = 5. Significance differences were assessed via one-way ANOVA: ### *p* < 0.001 vs. the control group; ** *p* < 0.01 and *** *p* < 0.001 vs. the STC group.

The protein levels of the GPR43 (~55 kDa) and 5-HT4 receptor (~55 kDa) in the colon of STC mice were determined via Western blotting (Figure 8B). In STC mice, the expressions of the GPR43 and 5-HT4 receptor were reduced (Figure 8B–D). Treatment with XCQ or mosapride recovered the STC-reduced protein expressions, i.e., those of the GPR43 (*p* < 0.05) and 5-HT4 receptor (*p* < 0.05), as compared to those in the STC group (Figure 8B–D). Additionally, the immunohistochemical results showed a significant reduction in the protein expressions of the GPR43 (*p* < 0.05) (Figure 9A,B) and 5-HT4 receptor (*p* < 0.001) (Figure 10A,B) in the colon of STC mice. As compared to the STC group, XCQ administration significantly raised the protein expressions of the GPR43 (*p* < 0.01) (Figure 9B) and 5-HT4 receptor (*p* < 0.001) (Figure 10B). The effects of XCQ were robust and as sound as those of mosapride.

## 3. Materials and Methods

### 3.1. Chemicals and Reagents

HPLC-grade acetonitrile, methanol, and formic acid were obtained from Merck (Darmstadt, Germany). Millipore Milli-Q water system supplied 18 MΩ cm^−1^ deionized water (Milford, MA, USA). Other reagents were of analytical purity. Short-chain fatty acids (acetic acid, propionic acid, isobutyric acid, butyric acid, and valeric acid) and an internal standard (2-ethylbutyric acid, IS) were purchased from Sigma-Aldrich, (purity > 98%, St. Louis, MO, USA). The BCA protein assay kit was purchased from Thermo Fisher Scientific (Rockford, IL, USA). RIPA lysis buffer was from Sigma-Aldrich. The secondary antibody was bought from Cell Signaling Technology (# 7074, # 7076, Danvers, MA, USA). The primary antibody for GPR43 was purchased from Proteintech (# 19952-1-AP, Wuhan, China). The primary antibody to the 5-HT4 receptor was purchased from Bioss Antibodies (# bs-2127R, Beijing, China). The primary antibody for GAPDH was bought from Sigma-Aldrich (# G8795). ECL was bought from Thermo Fisher scientific. The ELISA kit for 5-HT was bought from Jianglaibio (# JL12087, Shanghai, China). Rhei Radix et Rhizoma, Magnoliae Officinalis Cortex, and Aurantii Fructus Immaturus were provided by Guangdong Efong Pharmaceutical (Guangzhou, China). Loperamide hydrochloride (purity > 98%) was obtained from Sigma-Aldrich. Mosapride citrate was purchased from Yabao Pharmaceutical Group Co., Ltd. (Yuncheng, China).

### 3.2. Preparation of XCQ

The ground powders of herbal materials, composed of Rhei Radix et Rhizoma (Rhizomes of *R. palmatum*) 55.2 g, Magnoliae Officinalis Cortex (Barks of *M. officinalis*) 27.6 g, and Aurantii Fructus Immaturus (Fruitlet of *C. aurantium*) 36.0 g, in accordance with the traditional dosage of this herbal mixture, were immersed in 8 volumes of water (1:8, *w*/*v*) for 45 min before being extracted twice by boiling, then placed under gentle heating (300 W) until the liquid reached 240 mL. The decoction was filtered through 8 layers of gauze. The combined herbal extract was evaporated under a vacuum and lyophilized. The samples were stored at 4 °C.

### 3.3. Qualitative Analysis of XCQ Extract via HPLC

The HPLC analyses of XCQ were conducted by comparing the retention time of peaks with chemical standards. Methanol was added to make the mixture of standards (purity > 98%), containing naringin, neohesperidin, honokiol, magnolol, rhein, aloe-emodin, emodin, chrysophanol, and physcion, obtained from Weikeqi Biological Technology Co., Ltd. (Chengdu, China). Briefly, 20 mg of XCQ was dissolved in 1 mL of 80% methanol and analyzed via HPLC-DAD with a Waters CORTECS T3 column (2.1 mm × 150 mm, 1.6 µm). The injected volume was set as 1 µL. Solvents were as follows: solvent A, 0.1% diluted aqueous phosphoric acid, and solvent B, methanol. The elution procedure was as follows: solvent B increased from 3% to 21% at 0 to 5 min; 21% to 36% at 5 to 20 min; 36% to 50% at 20 to 32 min; 50% to 62% at 32 to 42 min; 62% to 85% at 42 to 50 min; 85% to 95% at 50 to 60 min. The column temperature was 30 °C, and the absorbance at 260 nm was collected. 

### 3.4. Experimental Environment

Balb/c mice (5–6 weeks old with a body weight of 20 ± 2 g) were purchased from Guangdong Medical Laboratory Animal Center. Mice were kept under specific pathogen-free conditions at Laboratory Animal Center, Kangmeihuada Gene Technology (Shenzhen, China), with 44–65% humidity and a 12-h light/dark cycle at 22–26 °C, and free access to food and water. The experimental protocols were approved and followed the guidelines of Chinese legislation on the ethical use and care of laboratory animals. 

### 3.5. Animal Experimental Design

After adjusting to the environment for 7 days, 84 pathogen-free mice were utilized to examine the preventative effects of XCQ on STC. The methods and procedures used in this animal experiment were performed in accordance with the Guidelines for the Care and Use of Laboratory Animals of The Hong Kong University of Science & Technology, reviewed and approved by the Animal Ethics Committee of Experimental Animals of The Hong Kong University of Science & Technology. The mice were randomly divided into six groups (*n* = 14): the control group, STC group, mosapride (a positive control and a gastroprokinetic agent acting as a selective 5-HT4 receptor agonist) group, XCQ_LD_ (low dose of XCQ 2 g/kg/day) group, XCQ_MD_ (medium dose of XCQ 4 g/kg/day) group, and XCQ_HD_ (high dose of XCQ 8 g/kg/day) group. Mice in the STC, mosapride, XCQ_LD_, XCQ_MD_, and XCQ_HD_ groups were treated with loperamide (10 mg/kg/day) via oral gavage twice a day for 7 days. The XCQ_LD_, XCQ_MD_, and XCQ_HD_ groups received varying doses of XCQ extract, whereas the mosapride group received mosapride (2.5 mg/kg/day) via oral gavage one hour after each dose of loperamide for 14 days. The mice in the control group were treated with the same volume of saline solution via oral gavage twice a day for 21 days. Weekly records of body weight, moist stool, and dry stool weight were kept. After collecting the mice’s stools on day 21, the animals were euthanized, and plasma and distal colonic tissues (10 mm) were taken out for analysis. All samples were stored at −80 °C for further analysis.

### 3.6. Measurement of Constipation-Related Indicators and Pathological Analysis

On the 21st day of experimental treatment, the parameters of constipation, e.g., the water content of feces, small intestinal transit, and time for first black stool defecation, were examined as previously described [19,20]. The colon tissues from the mice were routinely dehydrated, embedded, sectioned, and stained with hematoxylin and eosin (H&E) after being fixed in 4% paraformaldehyde for 24 h. Under a microscope, the pathological histological changes were viewed and captured on a camera.

### 3.7. Quantification of Short-Chain Fatty Acids in Feces

Using gas chromatography (GC), the amount of SCFAs were determined in mouse stool samples. Stool samples weighing 50 mg were homogenized by adding 100 µL of ultrapure water. The supernatant was then centrifuged, and the sample solution was obtained by combining 90 µL of the supernatant with 10 µL of an internal standard (2-ethyl butyric acid). The supernatants were analyzed using an Agilent GC-FID machine, equipped with an Agilent DB-WAXERT column (30 m × 0.25 mm × 0.25 μm). Helium (flow rate: 1.8 mL/min, split ratio 20:1) was used as the carrier gas. The injection temperature was 250 °C, and the GC temperature program was as follows: an initial temperature of 50 °C was raised to 130 °C at a rate of 15 °C/min and then to 200 °C and maintained for 2 min. The concentrations of SCFAs were calculated using standard curves for acetic acid, propionic acid, butyric acid, isobutyric acid, and valeric acid.

### 3.8. Bacterial Community Analysis Based on 16S rRNA Gene

The CTAB/SDS method was used to extract total DNA from the samples. DNA concentration and purity were measured, and diluted to 1 ng/µL using sterile water. The primers 515F (5′-GTG CCA GCM GCC GCG GTA A-3′) and 806R (5′-GGA CTA CHV GGG TWT CTA AT-3′) with the barcode were used to amplify the V4 region of bacterial the 16S ribosomal RNA gene. Sequencing libraries were generated using TruSeq^®^ DNA PCR-Free Sample Preparation Kit (Illumina, San Diego, CA, USA) following the manufacturer’s recommendations, and index codes were added. The library quality was assessed on Qubit@ 2.0 Fluorometer (Thermo Fisher Scientific) and the Agilent Bioanalyzer 2100 system. The library was sequenced on an Illumina NovaSeq platform, and 250 bp paired-end reads were generated. The raw reads from 16S sequencing were trimmed, filled, and merged using FLASH (version 1.2.11). Operational taxonomic unit (OTU) clustering was conducted through UCLUST at a 97% similarity level, and taxonomic assignment was performed through the RDP classifier with a minimal 95% confidence. α-diversity analysis was carried out in Mothur (version 1.30.2) with Shannon and Chao indexes. The rarefaction curve, community analysis, principal coordinates analysis (PCoA) and nonmetric multidimensional scaling (NMDS) were computed by R software (version 3.4.1). The linear discriminant analysis effect size (LefSe) was used as a non-parametric Wilcoxon sum-rank test, followed by LDA analysis to measure the effect size of each abundant taxon. 

### 3.9. Western Blotting Assay

Total proteins were extracted from the tissues using a lysis buffer, and the concentration was measured. Proteins were separated using SDS-PAGE and transferred to a nitrocellulose membrane, blocking with a 5% skim milk solution. Then, the relevant primary antibodies (GPR43 and 5-HT4 receptor) were incubated overnight on the membranes. The blots were washed three times before being exposed to the secondary antibody for an hour at room temperature. With the use of the improved chemiluminescent reagent (ECL), each antigen–antibody combination was discovered. The images were analyzed using Image-Pro Plus software version 1.53k (Media Cybernetics, Rockville, MD, USA).

### 3.10. Immunohistochemistry Assay

The expressions of the GPR43 and 5-HT4 receptor in colonic tissues were analyzed via by immunohistochemistry, as described in [13]. The slides were de-waxed with xylene, rehydrated, heated in a citrate buffer for antigen retrieval, incubated in 3% H_2_O_2_ for 5 min to quench endogenous peroxidase, blocked with goat serum for 15 min, incubated with primary antibodies (1:400) for 2 h at 37 °C, washed with PBS, incubated with secondary antibodies for 15 min at 37 °C, and finally developed with the DAB peroxidase (Thermo Fisher Scientific). After being counterstained with hematoxylin, the slides were mounted with neutral balsam, observed with a light microscope, and analyzed with Image-Pro Plus software (Media Cybernetics). In determining the level of 5-HT in plasma, the plasma was subjected to an ELISA kit accordingly, and the absorbance was set at 450 nm and measured on a microplate reader.

### 3.11. Statistical Analysis

Statistical analyses were performed via a one-way ANOVA with Tukey’s post hoc test using GraphPad Prism software Version 7 (GraphPad Software, La Jolla, CA, USA). Statistical significance was determined as (*) or (#) when *p* < 0.05.

## 4. Discussion

For thousands of years in China, numerous ailments have been prescribed for medical treatments using Chinese herbal medicine [21]. The XCQ decoction has been used clinically for thousands of years. In XCQ extract, HPLC analysis has identified nine major peaks, i.e., naringin, neohesperidin, honokiol, magnolol, rhein, aloe-emodin, emodin, chrysophanol, and physcion. The fingerprint could serve as a parameter for the quality control of XCQ, thereafter guaranteeing the repeatability of the experiments. Rhei Radix et Rhizoma contains active ingredients, e.g., chrysophanol, aloe-emodin, rhein, emodin, and physcion, showing different pharmacological activities, including purgative [22] and anti-inflammatory [23] ones. Aurantii Fructus Immaturus is a rich source of flavonoid-type compounds, such as naringin, neohesperidin, naringenin, and hesperetin; these flavonoids have diverse biological effects, including regulating gastrointestinal disorders [24]. In line with the function of XCQ, naringin is a potential natural agent that can protect mice suffering from ulcerative colitis [25]. The primary ingredients of Magnoliae Officinalis Cortex are magnolol and honokiol. The mixture of magnolol and honokiol has been shown to increase 5-HT in the prefrontal cortex of depressive mice, as well as regulating biochemical abnormalities of 5-HT and 5-hydroxyindoleacetic acid in the brain [26]. Thus, anthraquinones from Rhei Radix et Rhizoma, flavonoidic compounds from Aurantii Fructus Immaturus, and phenolic compounds from Magnoliae Officinalis Cortex could account for the components of the XCQ decoction that fight against constipation.

An over-the-counter anti-diarrheal drug, loperamide, causes difficulties in defecation by binding to the μ-opioid receptors in the intestinal circulation and longitudinal muscles. This increases the gastrointestinal transport time, slows down intestinal peristalsis, and inhibits the contraction of intestinal smooth muscle. The application of loperamide could lead to a decrease in intestinal transport and fecal water, and is being used to establish the animal model of STC. In this study, XCQ mediation was found to improve constipation-related parameters, constipation-associated colonic lesions, defecation, fecal water content, colonic mobility, and colonic lesions related to constipation in STC mice. These results suggest that XCQ could help in improving defecation and intestinal motility.

In line with the clinical outcome of XCQ mediation, the constipation-related parameters, and constipation-associated colonic lesions, identified in STC mice, were significantly recovered in mice treated with XCQ. In addition, defecation, fecal water content, colonic mobility, and colonic lesions relating to constipation were markedly improved. This result suggested that XCQ could intervene in defecation and intestinal motility.

Targeting 5-HT signaling is a recent approach for STC’s drug development. For example, mosapride, a gastroprokinetic agent acting as a selective 5-HT4 receptor agonist, has been marketed today to enhance gastrointestinal motility [27]; however, this drug has side effects of vomiting, drowsiness, dizziness, and headache. By activating various 5-HT receptors on the mucosal end of intrinsic primary afferent neurons, 5-HT triggers the peristaltic reflex [28]. The activated 5-HT receptors send signals to the myenteric plexus, which thereafter causes a wave of smooth muscle contraction in the intestine. The release of 5-HT by enterochromaffin cells impacts gut motility and intestinal microbiota [29]. Probiotics can increase intestinal output and hasten defecation by raising the amounts of SCFAs and 5-HT. In gut microbiota, *Bifidobacteria* and *Lactobacillus* spp. have been shown to increase the water content of stool in mice, which could be attributed to elevated fatty acid levels [20,30]. These microbes were shown here to be upregulated by XCQ treatment. 

Modulating the population of gut microbiota and the metabolism of SCFAs has emerged as a promising strategy for new drug development. The amount of *B. bifidum* in the gut could enhance the expression of the 5-HT4 receptor, thereby promoting colonic peristalsis and the secretion of intestinal fluid [31]. In response to SCFAs, the enterochromaffin cell releases 5-HT, which activates 5-HT3 receptors on vagal sensory fibers and causes muscular contraction [32]. The release of 5-HT from enterochromaffin cells was shown to be mediated by the activation of GPR43, a receptor for SCFAs [33]. These results imply that the profile of gut microbiota and the level of SCFAs are strongly correlated with 5-HT signaling. Supporting this notion, the treatment of XCQ could restore the loss of the 5-HT, GPR43, and 5-HT4 receptor and SCFAs in STC mice. Thus, XCQ in the gut can promote excitatory 5-HT signaling and positively enhance intestinal motor activity via the regulation of gut microbiota, which also suggests a possible relationship with the gut–brain axis.

This study has some limitations. Although 5-HT plays a crucial role in regulating bowel functions, the mechanistic action of 5-HT in gut function is complex. 5-HT induces smooth muscle contraction or relaxation through various subtypes of receptors. The activation of cholinergic excitatory neurons could be mediated by 5-HT3 and 5-HT4 receptors, and nitric oxide-inhibited enteric motor neurons could be mediated by 5-HT4, 5-HT1A, and 5-HT1D receptors, leading to a relaxation of the distal colon. Intestinal bacteria may lead to the upregulation of serotonin-selective reuptake transporter (SERT) expression, and as a result cause a decrease in 5-HT concentrations in colonic mucosa. In addition, some researchers have reported that increased 5-HT concentrations in the blood or colonic mucosa were closely related to constipation [34,35]. In addition, deficiency in 5-HT may cause delayed gastric emptying [36]. The total level of 5-HT can be affected by tryptophan hydroxylase (Tph1) and SERTs [37]. Tph1 is responsible for producing 5-HT in enterochromaffin cells, and then enters enterocytes or blood via SERTs. To fully understand the effects of XCQ in gut functions, further research is needed to study the effects of XCQ on the 5-HT level in the colon, as well as its effects on the expressions of Tph1 and SERTs.

## 5. Conclusions

This study focuses on the therapeutic role and mechanistic action of the XCQ herbal decoction in treating STC. XCQ exhibits an anti-constipation effect in loperamide-induced STC mice. Under the influence of XCQ in STC mice, the population of gut microbiota, the levels of SCFAs, the plasma level of 5-HT, and the expressions of the GPR43 and 5-HT4 receptor in the colon are restored back to those under a normal situation. Our results indicate that XCQ is a potent natural product that could be a therapeutic strategy for treating constipation. The pharmacological studies of natural products were conducted from 2022 to 2023. The authors are listed in order of their respective contributions to the work conducted during the study. 

## Data Availability

Data is contained within the article and supplementary material.

## Supplementary Materials

The following supporting information can be downloaded at https://www.mdpi.com/article/10.3390/ph17020153/s1: Figure S1: HPLC fingerprint of XCQ; Figure S2: The effects of XCQ on weekly body weight and water content of feces in STC mice; Figure S3: Refraction curves and α-diversity indexes of 16S rRNA sequencing.
