# Supplementary material for: Xiao Cheng Qi Decoction, an Ancient Chinese Herbal Mixture, Relieves Loperamide-Induced Slow-Transit Constipation in Mice: An Action Mediated by Gut Microbiota
Source: Pharmaceuticals (Basel). 2024 Jan 24;17(2):153. doi: 10.3390/ph17020153 (PMC10892578; doi:10.3390/ph17020153)

**Fig. S1.** HPLC fingerprint of XCQ.

The dried herbal extract of XCQ at 20 mg/mL was analyzed by HPLC chromatogram with a DAD detector at an absorbance of 260 nm. The injected volume was 1 $\mu$L. The peaks corresponding to anaringin, neohesperidin, honokiol, magnolol, rhein, aloe-emodin, emodin, chrysophanol, and physcion are indicated.

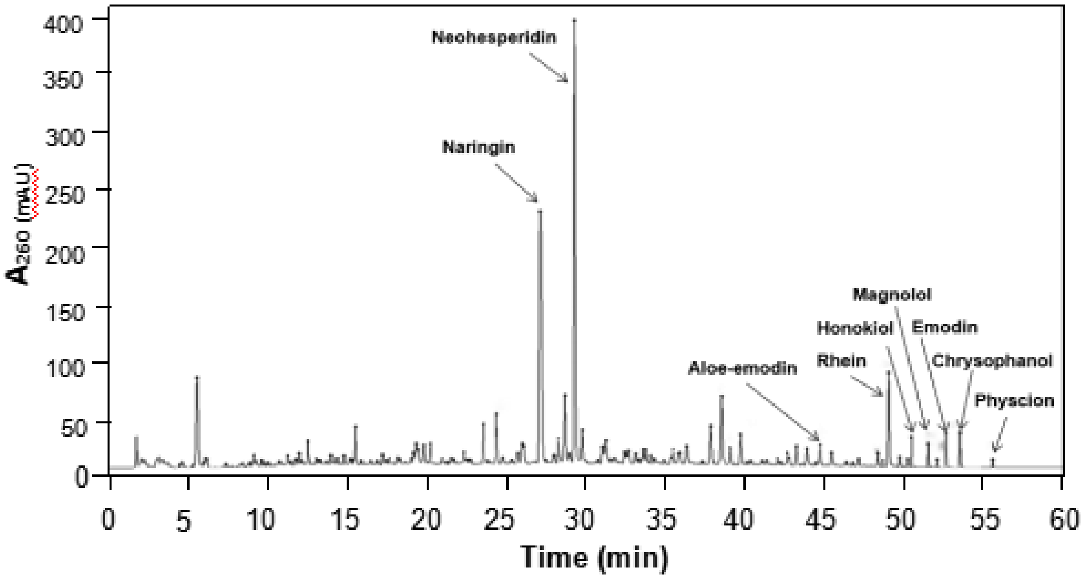

**Fig. S2.** The effects of XCQ on weekly body weight and water content of feces in STC mice.

Treatment of the mice was as in Fig. 1. Mouse body weight and fecal water content were determined at days 0, 7, 14 and 21, as indicated, during the experiment. **(A)**: The change of body weight ($n$ = 5). **(B)**: Water content of 50 mg of feces ($n$ = 3); Values are presented as mean ± SEM. Significance difference was assessed by one-way ANOVA: # $p < 0.05$, ## $p < 0.01$, ### $p < 0.001$ vs Control group; * $p < 0.05$, ** $p < 0.01$, *** $p < 0.001$ vs STC group.

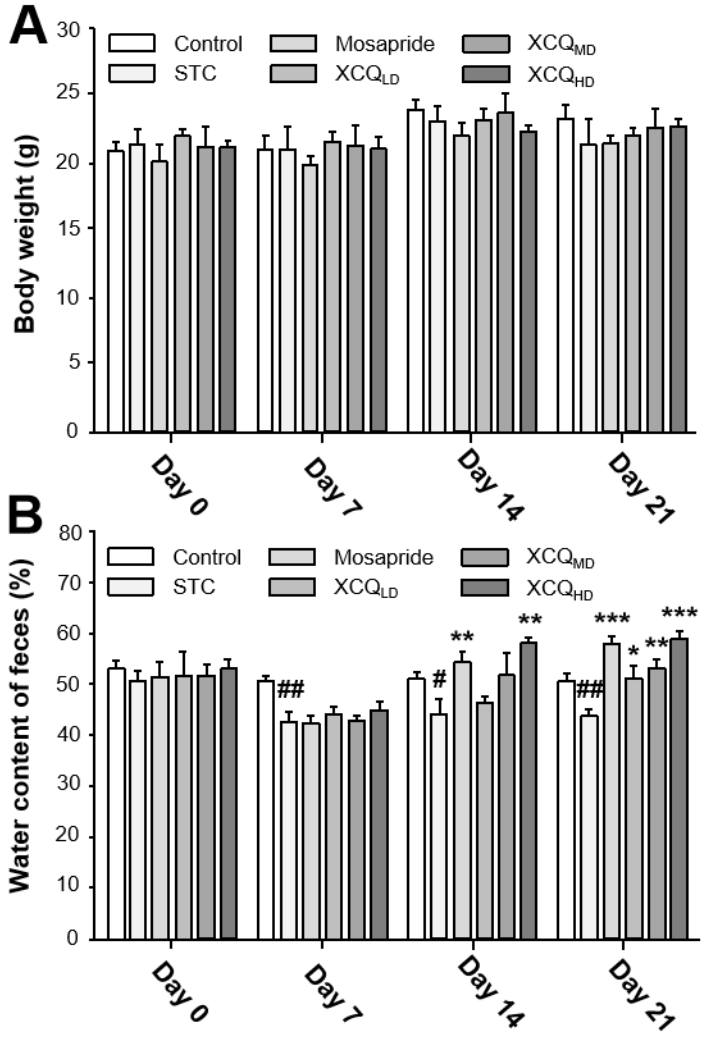

**Fig. S3.** Refraction curves and α-diversity indexes of 16S rRNA sequencing. **(A & B):** Refraction curves of each sample based on Shanno index (upper panel) and Chao index (lower panel) at OTU levels. $n = 4$. **(C & D):** $\alpha$-diversity indexes, i.e., Chao and Shannon indexes, stand for the richness and the diversity of each group. $n = 4$.

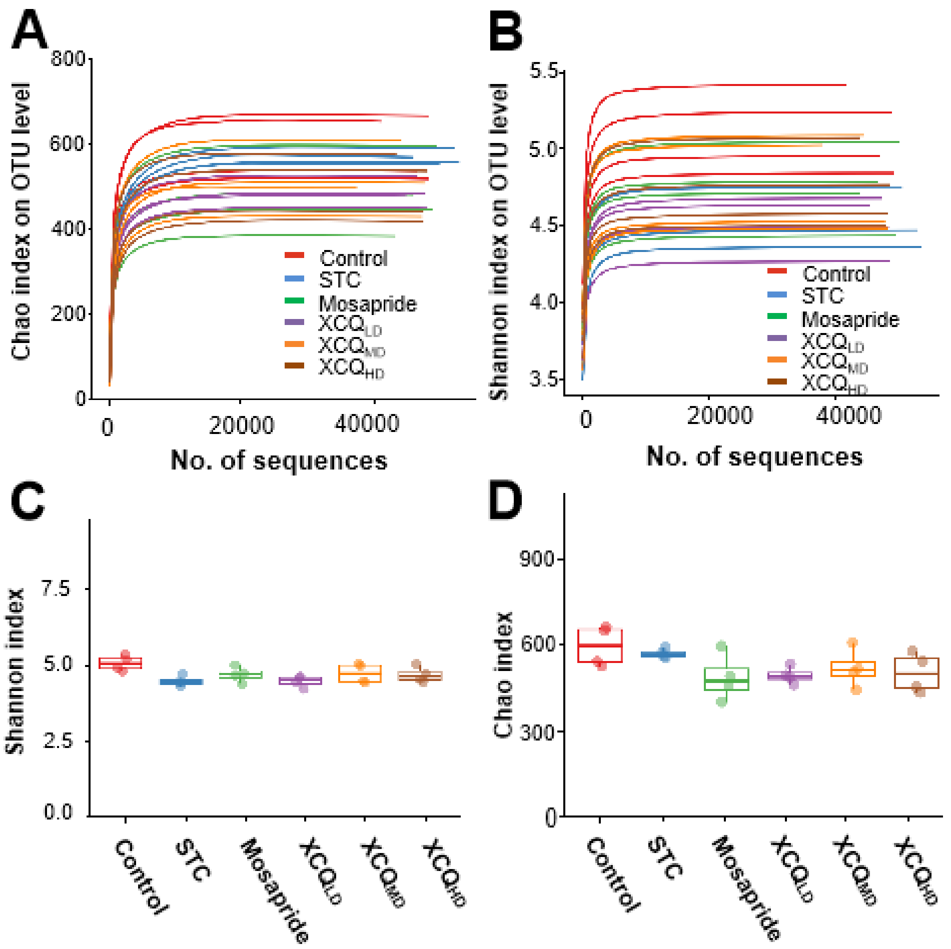

Supplement: Supplementary file 1 [file pharmaceuticals-17-00153-s001.zip › pharmaceuticals-2767813-supplementary.pdf]
